# Aberrant phosphorylation of human LRH1 at serine 510 is predictable of hepatocellular carcinoma recurrence

**DOI:** 10.1007/s10238-023-01098-x

**Published:** 2023-06-07

**Authors:** Atsushi Nishimagi, Makoto Kobayashi, Kotaro Sugimoto, Yasuhide Kofunato, Naoya Sato, Junichiro Haga, Teruhide Ishigame, Takashi Kimura, Akira Kenjo, Yasuyuki Kobayashi, Yuko Hashimoto, Shigeru Marubashi, Hideki Chiba

**Affiliations:** 1 Department of Hepato-Biliary-Pancreatic and Transplant Surgery, Fukushima Medical University School of Medicine, Fukushima, 960-1295 Japan; 2 Department of Basic Pathology, Fukushima Medical University School of Medicine, Fukushima, 960-1295 Japan; 3 Department of Diagnostic Pathology, Fukushima Medical University School of Medicine, Fukushima, 960-1295 Japan

**Keywords:** Biomarker, Liver cancer, Nuclear receptor, NR5A2, Prognosis

## Abstract

We previously identified the AKT-phosphorylation sites in nuclear receptors and showed that phosphorylation of S379 in mouse retinoic acid γ and S518 in human estrogen receptor α regulate their activity independently of the ligands. Since this site is conserved at S510 in human liver receptor homolog 1 (hLRH1), we developed a monoclonal antibody (mAb) that recognized the phosphorylation form of hLRH1S510 (hLRH1^pS510^) and verified its clinicopathological significance in hepatocellular carcinoma (HCC). We generated the anti-hLRH1^pS510^ mAb and assessed its selectivity. We then evaluated the hLRH1^pS510^ signals in 157 cases of HCC tissues by immunohistochemistry because LRH1 contributes to the pathogenesis of diverse cancers. The developed mAb specifically recognized hLRH1^pS510^ and worked for immunohistochemistry of formalin-fixed paraffin-embedded tissues. hLRH1^pS510^ was exclusively localized in the nucleus of HCC cells, but the signal intensity and positive rates varied among the subjects. According to the semi-quantification, 45 cases (34.9%) showed hLRH1^pS510^-high, and the remaining 112 cases (65.1%) exhibited hLRH1^pS510^-low. There were significant differences in the recurrence-free survival (RFS) between the two groups, and the 5-year RFS rates in the hLRH1^pS510^-high and hLRH1^pS510^-low groups were 26.5% and 46.1%, respectively. In addition, high hLRH1^pS510^ was significantly correlated with portal vein invasion, hepatic vein invasion, and high levels of serum alpha-fetoprotein (AFP). Furthermore, multivariable analysis revealed that hLRH1^pS510^-high was an independent biomarker for HCC recurrence. We conclude that aberrant phosphorylation of hLRH1S510 is a predictor of poor prognosis for HCC. The anti-hLRH1^pS510^ mAb could provide a powerful tool to validate the relevance of hLRH1^pS510^ in pathological processes such as tumor development and progression.

**Supplementary Information:**

The online version contains supplementary material available at 10.1007/s10238-023-01098-x.

## Background

Liver cancer is the sixth-most common malignancy and the third leading cause of cancer-related deaths worldwide [1]. Among liver cancer, hepatocellular carcinoma (HCC) accounts for approximately 85% of cases, and the major risk factors for HCC include chronic infection with hepatitis B virus or hepatitis C virus, alcohol consumption, and non-alcoholic fatty liver disease [2, 3]. There is a wide variety of therapeutic options for patients with HCC, such as hepatic resection, liver transplantation, ablation, intra-arterial embolization, radiotherapy, and systemic therapy [2–4]. However, HCC shows a high incidence of tumor recurrence, and it is reported that about 70% of patients with a single tumor in the early stages (Barcelona Clinic Liver Cancer [BCLC] stage 0 or A) after liver resection experience a recurrence within 5 years [5]. In addition, molecular classification of HCC has so far failed to predict disease progression, recurrence, or drug response [2, 6]. Furthermore, targeted therapy and chemotherapy exhibit limited efficiency in patients with advanced HCC [2–4, 7]. Therefore, novel biomarkers that predict HCC progression and relapse, as well as potential molecular targets to treat HCC, are urgently required.

Nuclear receptors are transcription factors that organize a broad range of physiological and pathological processes by regulating the expression of respective target genes [8, 9]. The nuclear receptor superfamily consists of 48 members in humans, and endogenous ligands have not been identified or established in more than half of nuclear receptors. The activity of nuclear receptors is controlled not only by ligand binding but also by post-translational modifications such as phosphorylation [10–13]. They also play an important role in tumor development and progression [14–17]. Additionally, nuclear receptors are promising targets for cancer treatment, and cancer treatment strategies targeting estrogen receptors (ERs) and retinoic acid receptors (RARs) have yielded significant results [17–19]. Moreover, a number of nuclear-receptor-based drugs, namely synthetic ligands, have been developed and entered clinical trials for various types of cancer [17, 19, 20].

The liver receptor homolog 1 (LRH1/NR5A2) is an orphan nuclear receptor and is expressed in a variety of organs, including the liver, pancreas, intestine, testis, ovary, uterus, and placenta, as well as the pituitary gland, hypothalamus, and adrenal gland [21–24]. It contributes to cholesterol homeostasis, steroidogenesis, reproduction, embryonic development, cell proliferation, and differentiation [22, 24, 25]. Endogenous ligands of LRH1 remain to be defined, even though several phospholipids are proposed to function as potential ligands [24, 26–28]. Concerning the phosphorylation of hLRH1, it is reported that S238 and S243 in the hinge domain can be phosphorylated by extracellular signal-regulated kinases (ERKs) [29]. S469 in isoform 2 of hLRH1 is also known to be phosphorylated by protein kinase A (PKA) [30]. Furthermore, LRH1 participates in the pathogenesis of numerous types of cancer [24, 31]. Among various cancer types, molecular biological approaches have shown that HCC progression is promoted by aberrant LRH1 expression [28, 32–35].

We previously identified the AKT-consensus phosphorylation motif (RXXS) in RARs and ERs, and found that phosphorylation of S379 in mouse RARγ and S518 in human ERα regulate their activity independently of their ligands [36, 37]. We subsequently demonstrated that ERαS518 is responsible for stimulation of the ERα activity in breast and endometrial cancer cells, as well as for endometrial cancer progression [38], indicating the functional relevance of this phosphorylation site, at least in these malignant cell types. Interestingly, these phosphorylation motifs are conserved in 14 of 48 members of human nuclear receptors [36], including human LRH1 (hLRH1). Taken together with the previous results showing that LRH1 drives liver cancer as described above [28, 32–35], we hypothesized that an aberrant hLRH1S510 phosphorylation (hLRH1^pS510^) affects the prognosis of HCC subjects.

In the present study, we developed a monoclonal antibody (mAb) that selectively recognizes hLRH1^pS510^ and works for immunohistochemistry of formalin-fixed paraffin-embedded (FFPE) tissues. Using this specific mAb, we showed that the high hLRH1^pS510^ represents a biomarker for HCC recurrence.

## Materials and methods

### Generation of antibodies

Rat mAbs against human LRH1^pS510^ were established using the iliac lymph node method [39]. Briefly, a polypeptide CPEIRAISMQAEEYL, in which S510 was phosphorylated, was coupled via the cysteine to Imject™ Maleimide-Activated mcKLH (77606; Thermo Fisher Scientific, Waltham, MA, USA). The conjugated peptide was intracutaneously injected with Dropper Adjuvant Complete Freund (263810; Becton Dickinson, Franklin Lakes, NJ, USA) into the footpads of anesthetized 8-week-old female rats. All animal experiments complied with the National Institutes of Health Guide for the Care and Use of Laboratory Animals and were approved by the Animal Committee at Fukushima Medical University (FMU) (approval code, 2021–092; approval date, May 10, 2021). The animals were sacrificed 14 days after immunization, and the median iliac lymph nodes were collected, followed by the extraction of lymphocytes by mincing. The extracted lymphocytes were fused with cells of the SP2 mouse myeloma cell line by polyethylene glycol. Hybridoma clones were maintained in GIT medium (637-25715; FUJIFILM Wako Pure Chemical, Osaka, Japan) with supplementation of 10% BM-Condimed (11088947001; Millipore Sigma, Burlington, MA, USA). The supernatants were screened by enzyme-linked immunosorbent assay (ELISA) and immunostaining. The specificity of the antibody was verified by antigen absorption and dephosphorylation assay. The complementarity-determinizing region (CDR) analysis was performed by Bio-Peak (Takasaki, Japan). Briefly, to determine the CDRs of the mAb, *V*_*H*_ and *V*_*L*_ regions were amplified by PCR with degenerate primers after mRNA extraction and reverse transcription. The amplicons were TA-cloned and sequenced.

### Cell Culture, expression vectors, and transfection

Human hepatocellular carcinoma cell lines HLF (JCRB0405) and JHH6 (JCRB1030) were purchased from JCRB cell bank (Osaka, Japan). Huh7.5.1 was gifted from Professor Matsuura, Osaka University. purchased from American Type Culture Collection. These cell lines were grown in Dulbecco’s Modified Eagle Medium (DMEM; D7777; Millipore Sigma, Burlington, MA, USA) with 10% fetal bovine serum (FBS; Millipore Sigma, Burlington, MA, USA) and 1% penicillin-streptomycin mixture (168-23191; FUJIFILM Wako Pure Chemical, Osaka, Japan). To overexpress human LRH1, the protein-coding region of *hLRH1* was cloned into the *NotI*/*BamHI* site of the CSII-EF-MCS-IRES2-Venus plasmid (RDB04384; Riken, Ibaraki, Japan). The hLRH1S510A mutant was established by a PCR-based standard mutagenesis protocol. The plasmids were transiently transfected to 293T cells using Polyethylenimine Max (24765-1; Polysciences, Warrington, PA, USA).

### Cell blocks

Cells were centrifuged at 1200 rpm for 10 min and fixed with 10% formalin for 16 h at 4 °C. Fixed cell pellets were mixed with 1% sodium alginate followed by 1 M calcium chloride and embedded in paraffin (Tissue-Tek VIP 5 Jr; Sakura Finetek Japan, Tokyo, Japan).

### ELISA

The antigen peptide and its unphosphorylated form were adsorbed onto Nunc-Immuno MaxiSorp plates (44-2404-21; Thermo Fisher Scientific, Waltham, MA, USA) overnight at 4 °C. After washing with Tris-buffered saline (TBS), non-specific reactivity was blocked by 1% Bovine Serum Albumin (BSA)/TBS for 30 min. The wells were incubated with the hybridoma supernatant for 1 h at 37 °C as the primary antibody. After washing with TBS, the plate was incubated with 2000 times diluted HRP-conjugated goat anti-rat antibody (NA935; Cytiva, Tokyo, Japan) for 1 h at 37 °C. 3,3′,5,5′ tetramethyl benzidine substrate kit (421101; Biolegend, San Diego, CA) was used for the detection.

### Antigen absorption

One hundred μL of hybridoma supernatant was pre-absorbed with 50 ng of the antigen peptide overnight at 4 °C, followed by centrifugation at 15,000 rpm for 10 min. The supernatant was used as the primary antibody.

### Dephosphorylation assay

The phospho-specificity of anti-hLRH1^pS510^ mAb was verified by ELISA and immunocytochemistry, using lambda protein phosphatase (sc-200312; Santa Cruz Biotechnology, Dallas, TX, USA) and phosphatase inhibitor (PhosSTOP™; 04906845001; Millipore Sigma, Burlington, MA, USA). For ELISA, after the antigens were coated, 800 U of lambda protein phosphatase was treated with or without phosphatase inhibitor for 2 h at 37 °C. For immunohistochemistry, the sections were incubated with 800 U of a lambda protein phosphatase with or without phosphatase inhibitor for 1 h at 37 °C after the antigen retrieval.

### Tissue collection

FFPE tissue sections were obtained from 157 patients with hepatocellular carcinoma (Supplementary Table 1) who underwent hepatectomy between Jan 2005 and Jan 2017 at FMU Hospital or Iwase General Hospital. Detailed information, including postoperative pathology diagnosis reports, age, gender, liver cirrhosis, alcoholic liver disease, tumor markers (AFP [alpha-fetoprotein], PIVKA-II [protein induced by vitamin K absence or antagonist-II]), tumor number, tumor size, differentiation, stage (the Union for Internationalis Contra Cancrum [UICC] 8th), portal vein invasion, hepatic vein invasion, bile duct invasion, surgical margin, overall survival (OS) and recurrence-free survival (RFS), was obtained. Distant metastasis was judged by diagnostic imaging. Five specimens of normal adult liver were collected from autopsy cases dissected at FMU Hospital between Jan 2018 and Dec 2019 (a 78-year-old female, a 63-year-old female, a 79-year-old female, a 71-year-old female, and a 67-year-old male). The study was approved by the Ethics Committee of FMU Hospital (approval code, 2020-058; approval date, Mar 16, 2021).

### Immunostaining and analysis

FFPE tissue sections from the cell blocks and HCC subjects were deparaffinized with xylene and rehydrated using a graduated series of ethanol. They were immersed in 0.3% hydrogen peroxide in methanol for 20 min at room temperature to block endogenous peroxidase activity. Antigen retrieval was performed by incubating the sections in boiling citric acid buffer (pH 6.0) in a microwave. After cooling at room temperature for 30 min, the sections were blocked with 1% BSA for 30 min. After blocking, the sections were incubated overnight at 4 °C with the primary antibodies. The VECTASTAIN Elite ABC HRP Kit for rat (PK-6104; Vector Laboratories, Burlingame, CA, USA) was used for 3′,3′-diaminobenzidine (DAB; 347-00904; DOJINDO, Kumamoto, Japan) staining.

Immunostaining results were interpreted by three independent pathologists and one gastroenterological surgeon using a semi-quantitative scoring system (immunoreactive score; IRS) (Supplementary Table 2) [40]. The immunostaining reactions were evaluated according to signal intensity (SI: 0, no stain; 1, weak; 2, moderate; 3, strong) and percentage of positive cells (PP: 0, < 1%; 1, 1–10%; 2, 11–30%; 3, 31–50%; and 4, > 50%). The SI and PP were then multiplied to generate the IRS for each case. Based on this analysis, we divided the samples into two groups based on the results of the immunostaining in the tissues: low expression (IRS ≦ 6) and high expression (IRS ≧ 8).

### Statistical analysis

We used the chi-squared test to evaluate the relationship between LRH1^pS510^ expression and various clinicopathological parameters (age, gender, liver cirrhosis, tumor markers [AFP, PIVKA-II], tumor number, differentiation, stage [UICC 8th], portal vein invasion, hepatic vein invasion, bile duct invasion, surgical margin, 5-year OS, and 5-year RFS). Survival analysis was performed using the Kaplan-Meier method, and differences between the groups were analyzed using the log-rank test. The Cox regression multivariable model was used to detect the independent predictors of survival. Two-tailed *P*-values < 0.05 were considered to indicate a statistically significant result. All statistical analyses were performed using SPSS version 26.0 software (IBM).

## Results

### Establishment of a mAb that selectively recognizes human LRH1^pS510^

**Fig. 1:**
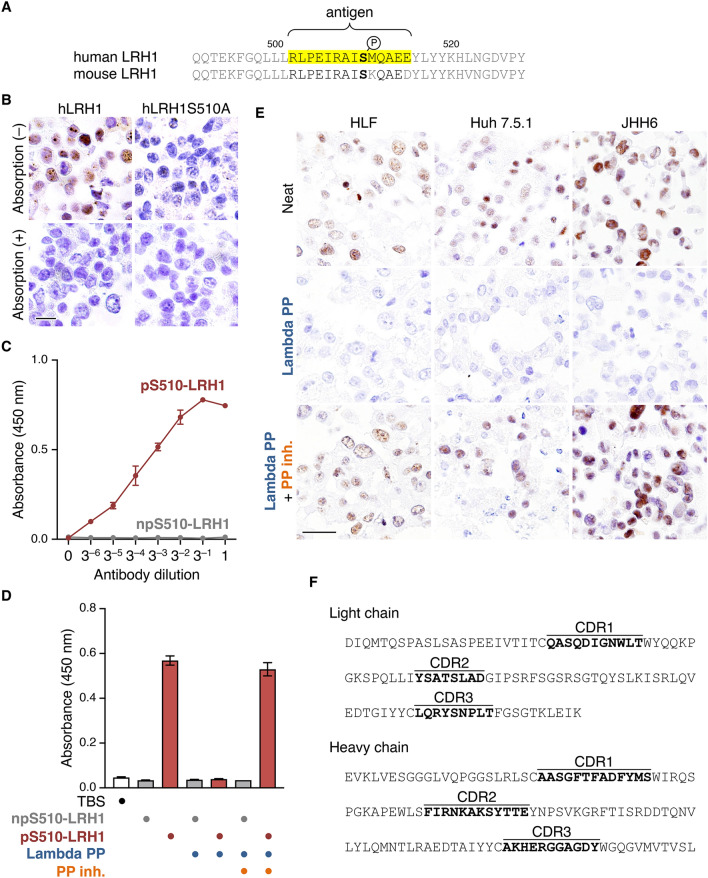
Generation of a rat mAb against human LRH1^pS510^. (**A)** Amino acid sequences of a part of the ligand-binding domain of human and mouse LRH1. The region that corresponds to an antigen peptide is highlighted. (**B)** Immunohistological images of hLRH1pS510 in 293T cells overexpressing hLRH1 or hLRH1S510A. Cell blocks of the transfected 293T were subjected to immunohistochemical analysis for hLRH1pS510 with or without antigen absorption. (**C)** ELISA analysis showing that an anti-hLRH1^pS510^ mAb (clone #55) reacts with the pS510-LRH1 polypeptide in dose-dependent manner. (**D)** ELISA analysis revealing that the selectivity of the anti-hLRH1^pS510^ mAb. TBS, Tris-buffered saline; Lambda PP, Lambda protein phosphatase; PP inh., Phosphatase inhibitor. (**E)** The hLRH1^pS510^ signal in the indicated hepatocellular carcinoma cells. The specificity of the anti-hLRH1^pS510^ is validated by immunohistochemistry of cell sections treated with Lambda PP alone or together with PP inh. (**F)** The complementarity-determining regions (CDRs) of the anti-hLRH1^pS510^ mAb (clone #55). Scale bars, 50 µm.

We generated an anti-human LRH1^pS510^ mAb using a pS510-LRH1 polypeptide, which contains phosphorylated S510, as an antigen (Fig. 1A). Upon screening by ELISA, 76 of 149 hybridomas showed both over 0.3 of absorbance against the pS510-LRH1 and under 0.3 of absorbance against the non-phosphorylated npS510-LRH1 polypeptides (Supplementary Fig. S1). We subsequently verified whether these 76 clones were able to detect signals by immunohistochemistry using cell blocks of 293T expressing wild-type hLRH1 or hLRH1S510A, in the latter of which hLRH1S510 was substituted for an alanine residue, with or without antibody absorption. Among the candidates, only clone #55 was able to detect nuclear signals in hLRH1-expressing 293T cells, and the positive signals disappeared upon antibody absorption (Fig. 1B). On the other hand, it failed to detect any positive signals in hLRH1S510A-expressing 293T cells. Therefore, we selected clone #55 of anti-LRH1^pS510^ mAb for further analysis.

The established anti-hLRH1^pS510^ mAb dose-dependently reacted with the pS510-LRH1 polypeptide, but did not recognize the non-phosphorylated one (Fig. 1C). In addition, binding of the anti-hLRH1^pS510^ mAb to pS510-LRH1 was completely prevented in the presence of lambda protein phosphatase, and the inhibition was reversed by phosphatase inhibitor (Fig. 1D). We also showed that the hLRH1^pS510^ signal was detected in three human HCC cell lines HLF, Huh7.5.1, and JHH6, by immunohistochemical analysis using their cell blocks (Fig. 1E). The specificity of the anti-hLRH1^pS510^ mAb was further confirmed by immunohistochemistry of these HCC cell sections treated with lambda protein phosphatase alone or together with phosphatase inhibitor. Moreover, we determined the CDR of the anti-hLRH1^pS510^ mAb (Fig. 1F).

### The hLRH1^pS510^ signals in HCC and non-tumor liver tissues

**Fig. 2:**
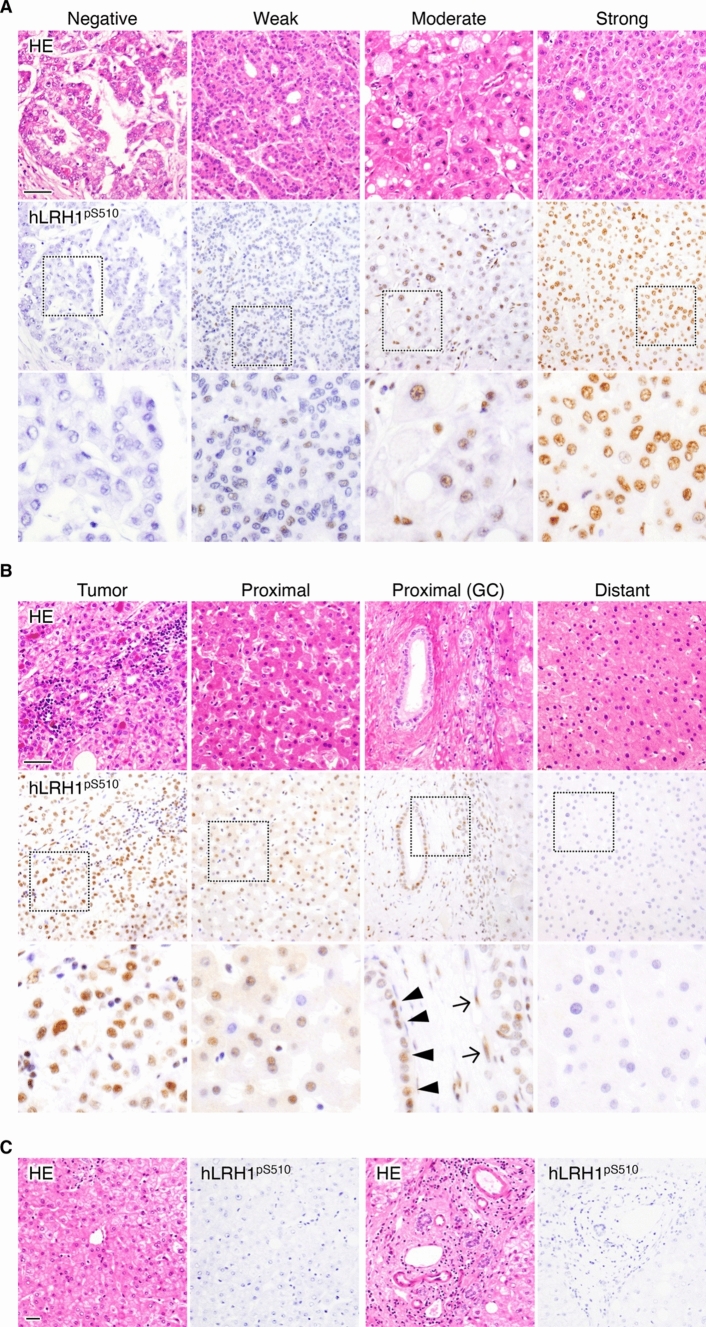
Representative immunohistological images of hLRH1^pS510^ in hepatocellular carcinoma (HCC) and non-tumor liver tissues. HCC (**A** and **B)** and non-neoplastic liver (**C)** tissues were immunohistochemically stained with the anti-hLRH1^pS510^ mAb. Arrowheads and arrows indicate positive nuclear signals in cholangiocytes and non-parenchymal cells close to the tumor possessing the hLRH1^pS510^-high signal, respectively. HE, hematoxylin-eosin; GC, Gleason’s capsule. Scale bars, 100 µm.

We next evaluated the hLRH1^pS510^ signals in 157 cases of human HCC tissues by immunohistochemistry. As shown in Fig. 2A and Supplementary Fig. S2, hLRH1^pS510^ appeared to be observed in the nuclei of HCC cells, but the signal intensity (SI) and percentage of positive cells (PP) varied among the subjects. Based on the semi-quantification using the immunoreactive score, 45 cases (34.9%) exhibited high hLRH1^pS510^, and the remaining 112 cases (65.1%) showed low hLRH1^pS510^ (Supplementary Fig. S3).

It should be noted that the moderate hLRH1^pS510^ signal was detectable in hepatocytes, cholangiocytes, and non-parenchymal cells close to the tumor possessing the hLRH1^pS510^-high signal (Fig. 2B, the middle two panels). On the other hand, no hLRH1^pS510^ signal was detected in the surrounding cells distant from the hLRH1^pS510^-high HCC, as well as in any cell types of human non-tumor liver tissues (Fig. 2B, C).

### hLRH1^pS510^-high correlates with recurrence and several clinicopathological factors in HCC

**Fig. 3:**
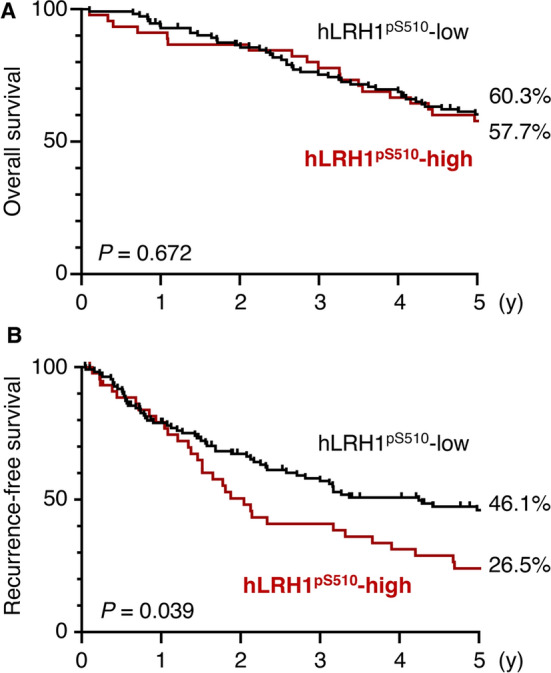
hLRH1^pS510^-high is associated with recurrence in hepatocellular carcinoma patients. The overall survival (**A**) and recurrence-free survival (**B**) for hLRH1^pS510^-high and hLRH1^pS510^-low in hepatocellular carcinoma subjects are indicated.

Kaplan-Meier plots revealed significant differences in RFS but not in OS between the LRH1^pS510^-high and LRH1^pS510^-low groups (Fig. 3A, B). The 5-year RFS rates in the LRH1^pS510^-high and LRH1^pS510^-low groups were 26.5% and 46.1%, respectively.

Among the clinicopathological variables, LRH1^pS510^-high was significantly associated with portal vein invasion (*P* = 0.008), hepatic vein invasion (*P* = 0.019), and high serum AFP (*P* = 0.027) (Table 1). By contrast, LRH1^pS510^-high was not associated with older age, male, liver cirrhosis, alcoholic liver disease, tumor number, tumor size (> 5 cm), tumor differentiation, bile duct invasion, positive surgical margin, stage II/IIIA/IIIB or serum PIVKA-II level.

**Table 1:** Relationship between the hLRH1^pS510^ signal and clinicopathological factors in patients with hepatocellular carcinoma (*n* = 157)

Parameter	Total		hLRH1^pS510^-low (*n* = 112)		hLRH1^pS510^-high (n = 45)		*P*-value
Age							0.366
< 65	41	(26.1)	27	(24.1)	14	(31.1)	
≧ 65	116	(73.9)	85	(75.9)	31	(73.9)	
Gender							0.648
Male	119	(75.8)	86	(76.8)	33	(73.3)	
Female	38	(24.2)	26	(23.2)	12	(26.7)	
Liver cirrhosis							0.763
(–)	104	(66.2)	75	(67.0)	29	(64.4)	
(+)	53	(33.8)	37	(33.0)	16	(35.6)	
Alcoholic liver disease							0.389
(–)	122	(77.7)	85	(75.9)	37	(82.2)	
(+)	35	(22.3)	27	(24.1)	8	(17.8)	
Tumor number							0.473
Single	127	(80.9)	89	(79.5)	38	(84.4)	
Multiple	30	(19.1)	23	(20.5)	7	(15.6)	
Tumor size							0.667
≦ 5 cm	112	(71.3)	81	(72.3)	31	(68.9)	
> 5 cm	45	(28.7)	31	(27.7)	14	(31.1)	
Differentiation							0.085
Moderate/poor	110	(70.1)	74	(66.1)	36	(80.0)	
Well	47	(29.9)	38	(33.9)	9	(20.0)	
Portal vein invasion							0.008
(–)	114	(72.6)	88	(78.6)	26	(57.8)	
(+)	43	(27.4)	24	(21.4)	19	(42.2)	
Hepatic vein invasion							0.019
(–)	140	(89.2)	104	(92.9)	36	(80.0)	
(+)	17	(10.8)	8	(7.1)	9	(20.0)	
Bile duct invasion							0.857
(–)	137	(87.3)	110	(98.2)	44	(97.8)	
(+)	20	(12.7)	2	(1.8)	1	(2.2)	
Surgical margin							0.218
(–)	143	(91.1)	104	(92.9)	39	(86.7)	
(+)	14	(8.9)	8	(7.1)	6	(13.3)	
Stage							0.059
IA/IB	95	(60.5)	73	(65.2)	22	(48.9)	
II/IIIA/IIIB	62	(39.5)	39	(34.8)	23	(51.1)	
AFP							0.027
≦ 10 ng/ml	69	(43.3)	55	(49.5)	13	(29.5)	
> 10 ng/ml	88	(56.1)	56	(50.9)	31	(70.5)	
PIVKA-II							0.73
≦ 40 mAU/ml	69	(43.9)	48	(43.2)	21	(46.7)	
> 40 mAU/ml	87	(55.4)	63	(56.8)	24	(53.3)	

Values are expressed as n (%).

### LRH1^pS510^-high is an independent prognostic marker for relapse of hepatocellular carcinoma

Univariable analysis revealed tumor number (hazard ratio [HR] = 1.847, 95% confidence interval [CI] 1.160–2.942, *P* = 0.010), tumor size (> 5 cm) (HR = 2.025, 95% CI 1.328–3.089, *P* = 0.001), portal vein invasion (HR = 1.741, 95% CI 1.135–2.669, *P* = 0.011), hepatic vein invasion (HR = 2.941, 95% CI 1.706–5.069, *P* < 0.001), stage II/IIIA/IIIB (HR = 2.196, 95% CI 1.464–3.293, *P* < 0.001), and LRH1^pS510^-high (HR = 1.555, 95% CI 1.018–2.374, *P* = 0.041), showed significant prognostic factors for the RFS of HCC patients (Table 2). In contrast, older age, male, liver cirrhosis, alcoholic liver disease, tumor differentiation, bile duct invasion, surgical margin, serum PIVKA-II level, or serum AFP level were not prognostic markers for HCC recurrence.

**Table 2:** Univariable analysis of recurrence-free survival in hepatocellular carcinoma patients

Variable		HR	95% CI	*P*-value
Age	≧ 65	1.406	0.878–2.252	0.156
Sex	Male	1.156	0.731–1.827	0.536
Liver cirrhosis	(+)	1.337	0.880–2.032	0.173
Alcoholic liver disease	(+)	1.007	0.620–1.635	0.978
Tumor number	Multiple	1.847	1.160–2.942	0.010
Tumor size	> 5 cm	2.025	1.328–3.089	0.001
Tumor differentiation	High	1.024	0.663–1.580	0.916
Portal vein invasion	(+)	1.741	1.135–2.669	0.011
Hepatic vein invasion	(+)	2.941	1.706–5.069	0.000
Bile duct invasion	(+)	1.037	0.255–4.212	0.960
Surgical margin	(+)	0.938	0.455–1.937	0.863
Stage	II/IIIA/IIIB	2.196	1.464–3.293	0.000
AFP	> 10 ng/ml	1.243	0.825–1.871	0.299
PIVKA-II	> 40 mAU/ml	1.459	0.966–2.202	0.072
hLRH1^pS510^	High	1.555	1.018–2.374	0.041

*HR,* hazard ratio; *CI,* confidence interval.

Cox multivariable analysis revealed that tumor number (HR = 1.958, 95% CI 1.224–3.131, *P* = 0.005), tumor size (HR = 1.725, 95% CI 1.08–2.753, *P* = 0.022), hepatic vein invasion (HR = 2.219, 95% CI 1.207–4.076, *P* = 0.010), and LRH1^pS510^-high (HR = 1.558, 95% CI 1.009–2.407, *P* = 0.046) were independent prognostic factors for the RFS of HCC subjects (Table 3).

**Table 3:** Multivariable analysis of recurrence-free survival in hepatocellular carcinoma patients

Variable		HR	95% CI	*P*-value
Tumor number	Multiple	1.958	1.224–3.131	0.005
Tumor size	> 5 cm	1.725	1.08–2.753	0.022
Hepatic vein invasion	(+)	2.219	1.207–4.076	0.010
hLRH1^pS510^	High	1.558	1.009–2.407	0.046

*HR,* hazard ratio; *CI,* confidence interval.

## Discussion

S469 in isoform 2 of hLRH1, which corresponds to S510 in isoform 1 of hLRH1, is known to be phosphorylated by PKA [30]. However, a lack of the phosphorylation-specific Ab hampers the verification of the significance of these phosphorylation sites in normal and pathological tissues. Therefore, in the present study, we established a novel mAb that selectively recognizes hLRH1^pS510^. The specificity of this mAb (clone #55) was confirmed by the following results: (1) on ELISA analysis, it reacted with the pS510-LRH1 polypeptide in a dose-dependent manner, whereas it did not respond to the non-phosphorylated one; (2) its binding to pS510-LRH1 was completely blocked by lambda protein phosphatase, and the inhibition was reversed upon addition of phosphatase inhibitor; (3) by immunohistochemistry, it was able to detect nuclear signals in hLRH1-expressing 293T cells but not in hLRH1S510A-expressing ones; (4) positive signals in hLRH1-expressing 293T disappeared upon antibody absorption; (5) immunohistochemical analysis further showed that positive nuclear signals in three human HCC cells were lost by lambda protein phosphatase and recovered together with the phosphatase inhibitor. Thus, owing to these high selectivities of the developed anti-hLRH1^pS510^ mAb and its application to immunohistochemistry of FFPE tissues, it could be a valuable tool to evaluate the importance of hLRH1^pS510^ in a variety of normal and pathological tissues.

By immunohistochemical analysis using the anti-hLRH1^pS510^ mAb, we demonstrated that the high hLRH1^pS510^ signal was observed in 45 of 157 HCC cases (34.9%). The positive hLRH1^pS510^ signals were exclusively restricted in the nuclei of HCC tissues. On the other hand, no hLRH1^pS510^ signal was distributed in non-tumor liver tissues, or in the tissues apart from HCC nests showed a high hLRH1^pS510^ signal. Interestingly, however, both parenchymal and non-parenchymal cells in close proximity to HCC tissues with hLRH1^pS510^-high also exhibited positive hLRH1^pS510^ signals to a moderate extent. Hence, the HCC tissues may release some factors, leading to the phosphorylation of hLRH1S510 in the surrounding non-tumor cells.

The most important conclusion in the present study is that aberrant phosphorylation of hLRH1S510 is able to predict the relapse of HCC. The RFS in the hLRH1^pS510^-high group of the HCC subjects was significantly lower than that in the hLRH1^pS510^-low group. Additionally, upon univariable analysis, high hLRH1^pS510^ exhibited a significant prognostic variable for the RFS of HCC patients. Moreover, multivariable analysis showed that hLRH1^pS510^-high was an independent prognostic marker for the RFS of HCC subjects. Furthermore, hLRH1^pS510^-high was also significantly associated with portal vein invasion and hepatic vein invasion, further suggesting the clinicopathological relevance of abnormal hLRH1S510 phosphorylation in HCC progression. Analysis of a large number of cases would be required to obtain more solid conclusions.

It is unknown by which mechanisms aberrant phosphorylation of S510 in hLRH1 could contribute to relapse, portal, and hepatic vein invasion in HCC subjects. However, hLRH1S510 is the AKT-consensus phosphorylation site as described above, and AKT is activated in many types of cancers, including HCC [3]. Therefore, it is reasonable that hLRH1S510 is phosphorylated by AKT in HCC tissues. In addition, we should mention our previous findings on the conserved AKT-phosphorylation sites, hERαS518 and mRARγS379. For example, we formerly reported that abnormal phosphorylation of S518 in hERα is indispensable for the regulation of target gene expression in breast and endometrial cancer cells, as well as for endometrial cancer progression such as cell proliferation and migration [36–38]. Furthermore, we previously showed that phosphorylation of mRARγS379 results in the dissociation of the nuclear receptor corepressor (NCoR) from RA response elements in the promoter of target genes, thereby activating their expression [36]. Thus, hLRH1^pS510^ possibly participates in HCC progression by a mechanism similar to hERα^pS518^ and mRARγ^pS518^ (Supplementary Fig. S4).

In summary, the present study demonstrated that abnormal hLRH1S510 phosphorylation predicted poor prognosis for patients with HCC. Further study is required to determine whether and how S510 phosphorylation of hLRH1 contributes to HCC progression, as well as the use of hLRH1^pS510^ as a potential therapeutic target for HCC.

### Supplementary Information

Below is the link to the electronic supplementary material.

Supplementary file1 (DOCX 5929 KB)

## Data Availability

All data generated or analyzed during this study are included in this article and its online supplementary material. Further enquiries can be directed to the corresponding author.

## Abbreviations

AFP: Alpha-fetoprotein
BCLC: Barcelona clinic liver cancer
BSA: Bovine serum albumin
CDR: Complementarity-determinizing region
CI: Confidence interval
ELISA: Enzyme-linked immunosorbent assay
ERKs: Extracellular signal-regulated kinases
ERs: Estrogen receptors
FFPE: Formalin-fixed paraffin-embedded
HCC: Hepatocellular carcinoma
HR: Hazard ratio
IRS: Immunoreactive score
LRH1: Liver receptor homolog 1
mAb: Monoclonal antibody
NCoR: Nuclear receptor corepressor
OS: Overall survival
PIVKA-II: Protein induced by vitamin K absence or antagonist-II
PKA: Protein kinase A
RARs: Retinoic acid receptors
RFS: Recurrence-free survival
TBS: Tris-buffered saline
