# Supplementary material for: Aberrant phosphorylation of human LRH1 at serine 510 is predictable of hepatocellular carcinoma recurrence
Source: Clin Exp Med. 2023 Jun 7;23(8):4985–95. doi: 10.1007/s10238-023-01098-x (PMC10725388; doi:10.1007/s10238-023-01098-x)
Supplement: Supplementary file 1 — Supplementary file1 (DOCX 5929 KB) [file 10238_2023_1098_MOESM1_ESM.docx]

Supplementary Information for

Aberrant phosphorylation of human LRH1 at serine 510 is predictable of hepatocellular carcinoma recurrence

Atsushi Nishimagi, Makoto Kobayashi, Kotaro Sugimoto, Yasuhide Kofunato, Naoya Sato, Junichiro Haga, Teruhide Ishigame, Takashi Kimura, Akira Kenjo, Yasuyuki Kobayashi, Yuko Hashimoto, Shigeru Marubashi, Hideki Chiba

Kotaro Sugimoto, Hideki Chiba

Email: [sugikota@fmu.ac.jp](mailto:sugikota@fmu.ac.jp) hidchiba@fmu.ac.jp

This file includes:

Figures S1 to S4

Tables S1 and S2

**
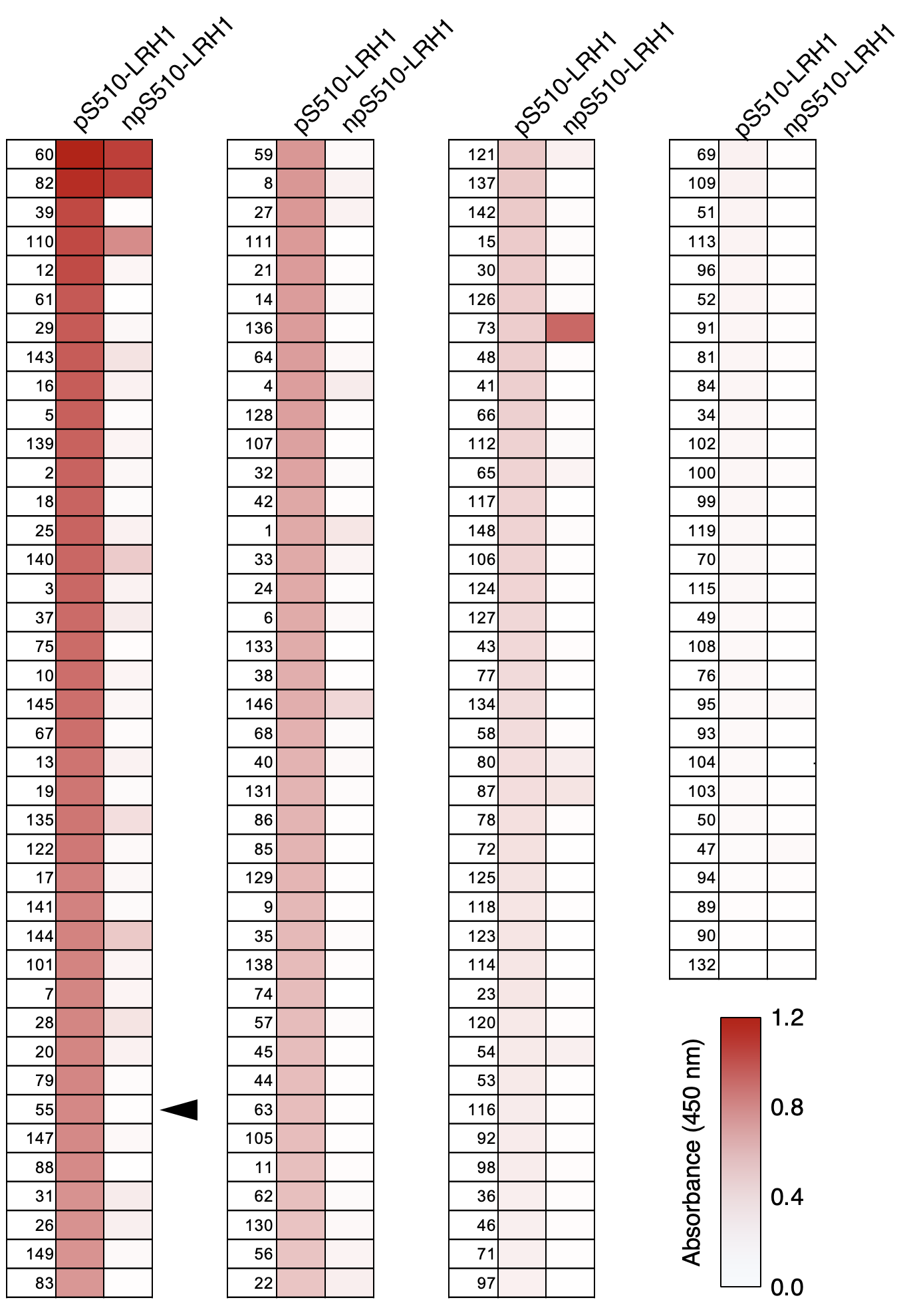
**

**Fig. S1.** ELISA analysis showing the binding of candidates for anti-hLRH1^pS510^ mAbs to the phosphorylated pS510-LRH and the non-phosphorylated npS510-LRH1 peptide. The absorbance (450 nm) levels are shown in the heatmap. The arrowhead indicates the selected clone of anti-hLRH1^pS510^ mAb.

**
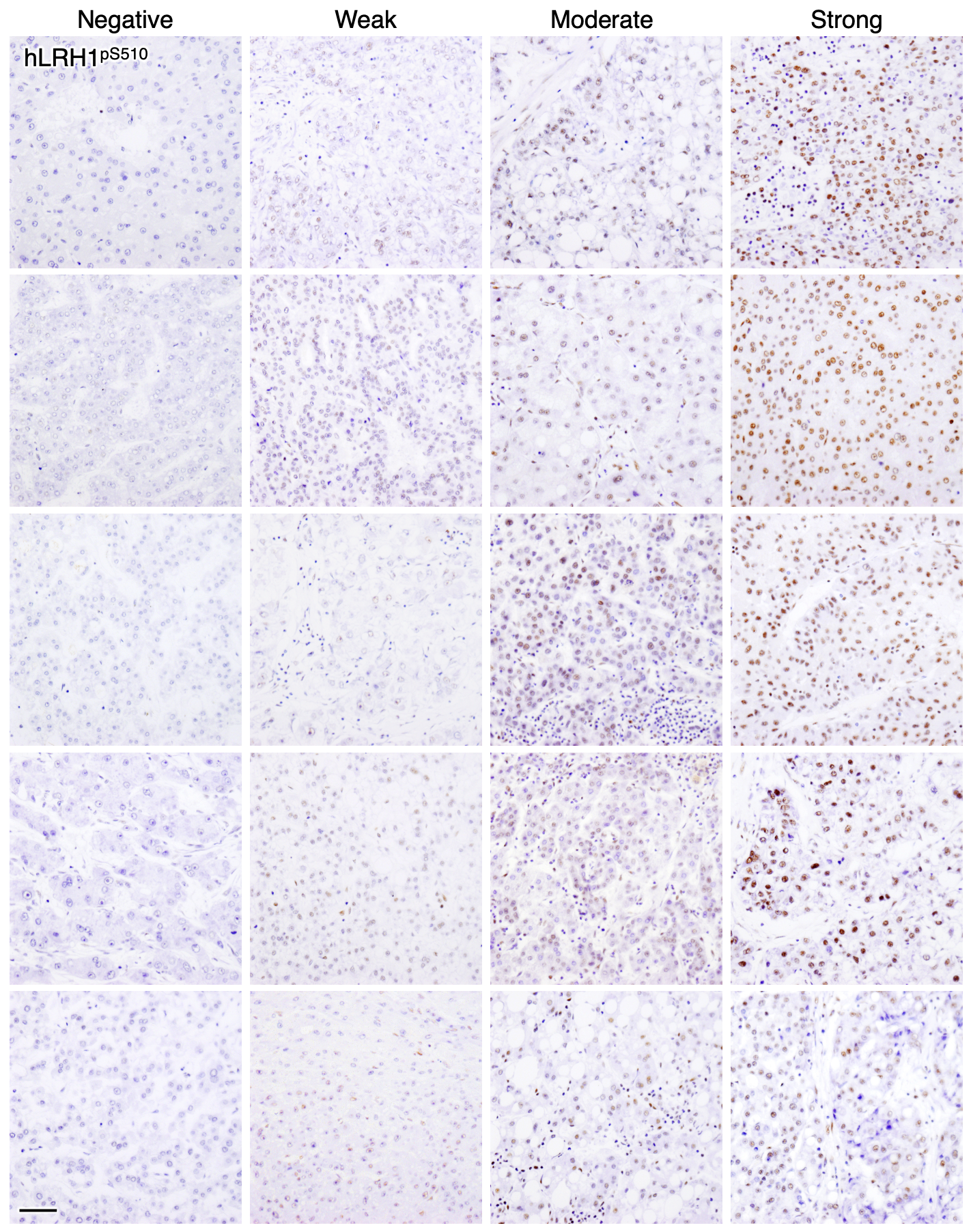
**

**Fig. S2.** Immunohistological images of hLRH1^pS510^ in hepatocellular carcinoma tissues. They were stained with the anti-hLRH1^pS510^ mAb, and representative images showing negative/weak/moderate/strong intensity for hLRH1^pS510^ in 20 cases of hepatocellular carcinoma tissues are indicated. Scale bar, 100 µm.


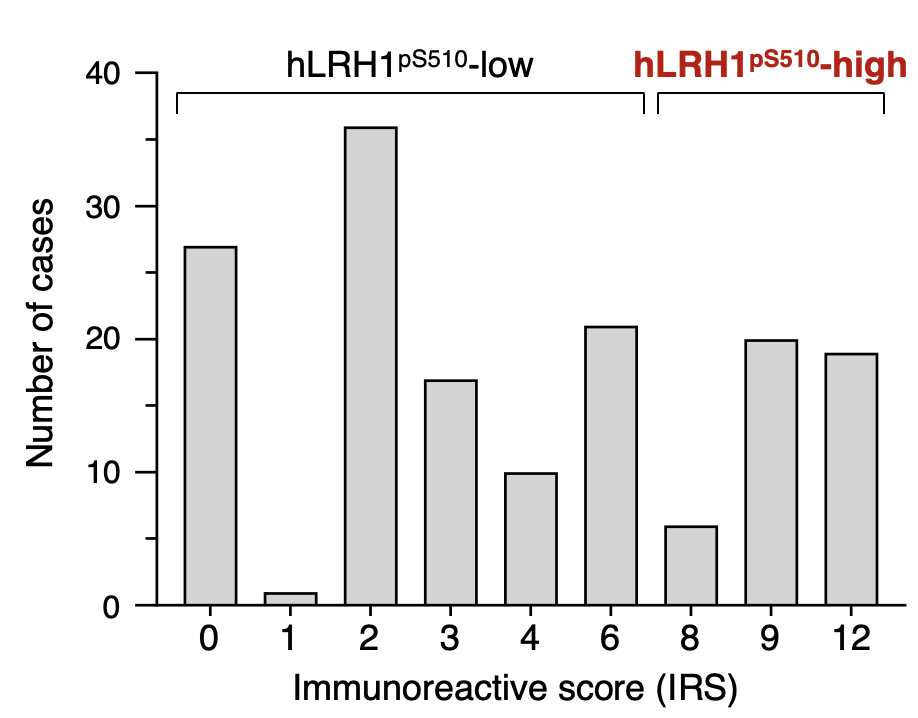


**Fig. S3.** Semi-quantification of the hLRH1^pS510^ signals in 157 cases of hepatocellular carcinoma tissues.

**
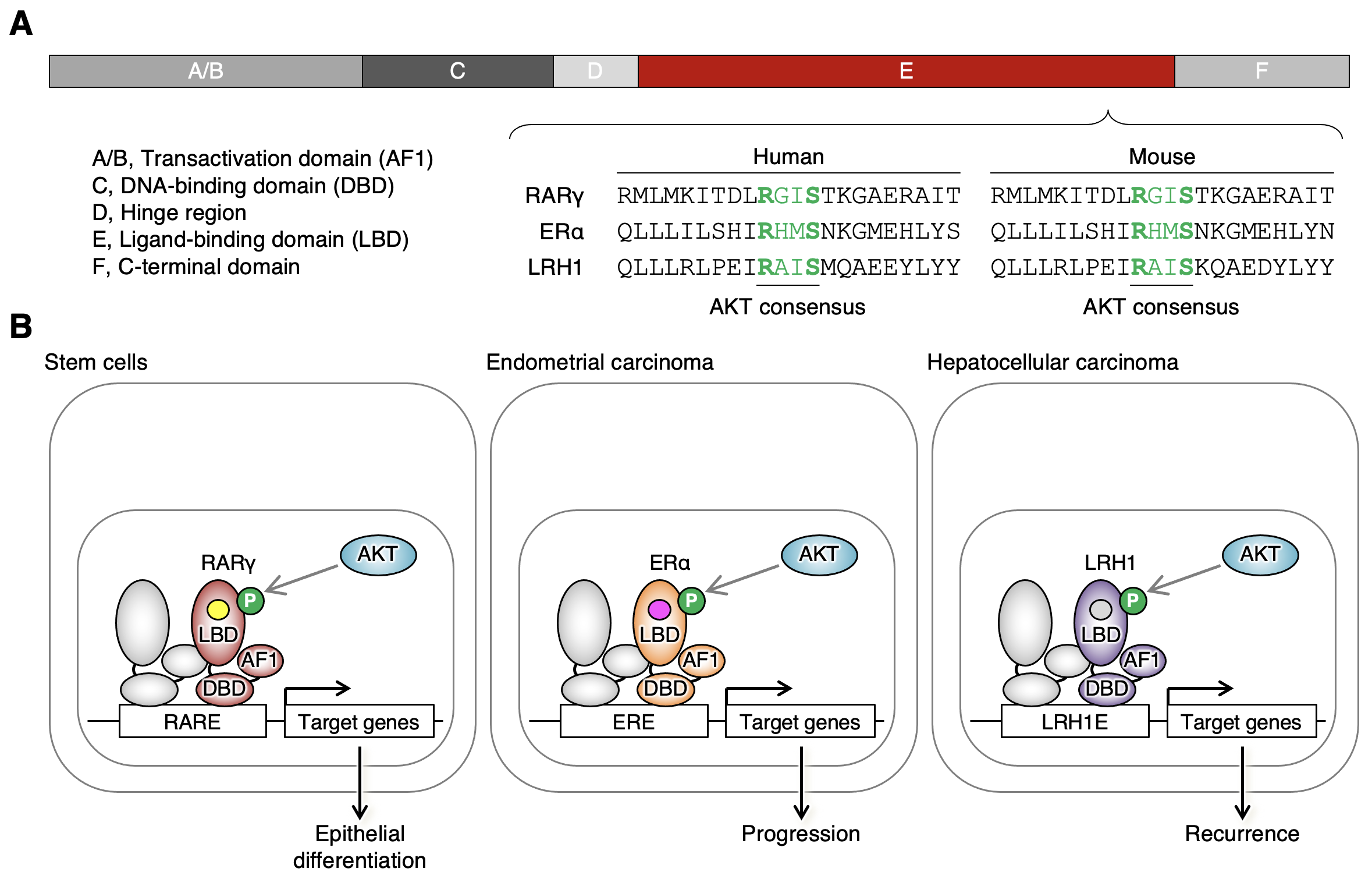
**

**Fig. S4.** Schematic model for regulation of the nuclear receptor activity by AKT. (A) Domain structure of nuclear receptors RARγ, ERα, and LRH1. The consensus AKT-phosphorylation motifs are indicated in green. (B) AKT-dependent phosphorylation of mRARγS379, hERαS518, and hLRH1S510 contribute to epithelial differentiation (Sugimoto et al., 2019), embryonal carcinoma progression (Kojima et al., 2021), and hepatocellular carcinoma recurrence (this study), respectively. Yellow circle, retinoic acid; pink circle, estrogen.

**Table S1.** Clinicopathological characteristics of patients with hepatocellular carcinoma (n=157).

| Parameter | | Value |  |
| --- | --- | --- | --- |
| Age | |  |  |
|  | years ± mean (range) | 71.0 ± 9.9 | (36–87) |
|  |  |  |  |
| Gender | |  |  |
|  | Male | 119 | (75.8) |
|  | Female | 38 | (24.2) |
|  |  |  |  |
| Stage | |  |  |
|  | IA/IB | 95 | (60.5) |
|  | II | 48 | (30.6) |
|  | IIIA/IIIB | 14 | (8.9) |
|  |  |  |  |
| Histopathological Tumor differentiation | |  |  |
|  | Well | 47 | (29.9) |
|  | Moderate | 102 | (65.0) |
|  | Poor | 8 | (5.1) |

Values are expressed as n (%).

**Table S2.** Immunoreactivity score (IRS) for hLRH1^pS510^.

| Score | Signal Intensity (SI) | Percentage of Positive Cells (PP) |
| --- | --- | --- |
| 0 | negative | <1% |
| 1 | weak | 1-10% |
| 2 | moderate | 11-30% |
| 3 | strong | 31-50% |
| 4 |  | >50% |
| SS×PP |  |  |
| 0 | Score0 | hLRH1^pS510^-low |
| 1-2 | Score1+ |  |
| 3-6 | Score2+ |  |
| 8-12 | Score3+ | hLRH1^pS510^-high |
